# Genetic and environmental influences on obsessive–compulsive behaviour across development: a longitudinal twin study

**DOI:** 10.1017/S0033291714002761

**Published:** 2014-12-12

**Authors:** G. Krebs, M. A. Waszczuk, H. M. S. Zavos, D. Bolton, T. C. Eley

**Affiliations:** 1 King's College London, MRC Social, Genetic and Developmental Psychiatry Centre, Institute of Psychiatry, Psychology and Neuroscience, De Crespigny Park, London, UK; 2 National and Specialist OCD and Related Disorders Clinic for Young People, South London and Maudsley NHS Foundation Trust, London, UK; 3 King's College London, Department of Psychology, Institute of Psychiatry, Psychology and Neuroscience, De Crespigny Park, London, UK

**Keywords:** Development, genetics, obsessive–compulsive disorder, paediatric research, twins

## Abstract

**Background:**

Little is known about the factors influencing the stability of obsessive–compulsive behaviour (OCB) from childhood to adolescence. The current study aimed to investigate: (1) the stability of paediatric OCB over a 12-year period; (2) the extent to which genetic and environmental factors influence stability; and (3) the extent to which these influences are stable or dynamic across development.

**Method:**

The sample included 14 743 twins from a population-based study. Parental ratings of severity of OCB were collected at ages 4, 7, 9 and 16 years.

**Results:**

OCB was found to be moderately stable over time. The genetic influence on OCB at each age was moderate, with significant effects also of non-shared environment. Genetic factors exerted a substantial influence on OCB persistence, explaining 59–80% of the stability over time. The results indicated genetic continuity, whereby genetic influences at each age continue to affect the expression of OCB at subsequent ages. However, we also found evidence for genetic attenuation in that genetic influences at one age decline in their influence over time, and genetic innovation whereby new genes ‘come on line’ at each age. Non-shared environment influenced stability of OCB to a lesser extent and effects were largely unique to each age and displayed negligible influences on OCB at later time points.

**Conclusions:**

OCB appears to be moderately stable across development, and stability is largely driven by genetic factors. However, the genetic effects are not entirely constant, but rather the genetic influence on OCB appears to be a developmentally dynamic process.

## Introduction

Obsessive–compulsive disorder (OCD) affects 1–4% of children and adolescents (Flament *et al.* 1988; Douglass *et al.* 1995; Heyman *et al.* 2001), and causes functional impairment in multiple domains (Piacentini *et al.* 2003). Although the disorder is often considered chronic and lifelong, there has been relatively little research into the stability of OCD in paediatric populations. The only meta-analysis to date of long-term outcomes in paediatric OCD included 521 participants from 16 studies (Stewart *et al.* 2004). A persistence rate of 41% for OCD and 60% for subclinical OCD symptoms was found over a mean follow-up period of 5.7 years. Similar persistence rates have been found in more recent studies (Micali *et al.* 2010; Mancebo *et al.* 2014). However, most studies to date have been limited by small sample sizes and short follow-up periods, and they typically focus on clinical cohorts rather than community samples (Stewart *et al.* 2004), and may therefore reflect the long-term effects of treatment rather than the natural course of the disorder.

A key question that has received little empirical attention is: what influences the persistence of OCD symptoms over time? Cross-sectional twin studies have found genetic factors to explain 45–65% of the variance of OCD symptoms at different ages during childhood and adolescence, with the remainder of the variance typically being explained by non-shared environmental influences (Eley *et al.* 2003; Hudziak *et al.* 2004; van Grootheest *et al.* 2005, 2008; Bolton *et al.* 2007; Hur & Jeong, 2008; Taylor, 2011). However, these studies do not inform us about the extent to which genetic and environmental factors drive stability of OCD symptoms over time.

To our knowledge, only two studies to date have investigated possible genetic and environmental contributions to OCD symptom stability, both using longitudinal twin designs (van Grootheest *et al.* 2007, 2009). The first explored stability of obsessive–compulsive behaviour (OCB) from ages 7 to 12 years (van Grootheest *et al.* 2007). OCB was found to be moderately stable over this interval and genetic factors explained approximately 40% of that stability, with the remaining variation accounted for by shared and non-shared environment. The second study examined the stability of OCB over an 11-year period among a cohort of young adults (van Grootheest *et al.* 2009). Again, they found a moderate level of phenotypic stability and genetic factors explained approximately 75% of OCB stability, with the remaining variance accounted for by non-shared environmental factors. Taken together, the results of these studies provide strong evidence that genetic factors influence the persistence of obsessive–compulsive symptomatology, but there might be developmental differences in the role of the environment. Moreover, it remains unclear whether these findings would extend to OCD symptom stability across a greater range of developmental stages, such as from early childhood through to adolescence. This is a period that is associated with significant biological and social changes, and a rising prevalence of OCD (Heyman *et al.* 2001).

Little empirical attention has been given to understanding the pattern of genetic risk for OCB across development. One possibility is that a single set of genes influences OCB at all ages. Alternatively, the effect of genes may vary over time, with different genetic factors influencing OCB at different stages of development. The two previous longitudinal twin studies of OCB provided clear evidence for genetic continuity but little evidence for genetic innovation (van Grootheest *et al.* 2007, 2009). This finding is in keeping with a number of other studies which suggest that genetic effects on child emotional development are largely stable and account for continuity in symptoms, whereas environmental influences tend to be time-specific and account for change in symptoms over time (O'Connor *et al.* 1998; Eaves & Silberg, 2008; Trzaskowski *et al.* 2012; Zavos *et al.* 2012). However, some studies have found evidence of genetic continuity but also significant genetic innovation and attenuation (Scourfield *et al.* 2003; Lau & Eley, 2006; Kendler *et al.* 2008*a*, *b*). For example, a study exploring genetic and environmental influences on mixed anxiety and depressive symptoms found that genetic factors influencing symptoms at age 8–9 years greatly declined in their effect by 19–20 years of age (i.e. attenuation), and new genetic influences emerged at later ages (i.e. innovation) (Kendler *et al.* 2008*a*). Different findings across studies may reflect methodological variations, including in the trait being measured and the time period over which is it assessed. It is possible that for some phenotypes genetic effects are stable over certain periods but not others. For example, genetic influences on depression may be dynamic during adolescence (Lau & Eley, 2006) but stable in adulthood (Kendler *et al.* 1993). This highlights the importance of examining the temporal stability of genetic effects across different stages of development.

In summary, given the prevalence and morbidity associated with paediatric OCD, a better understanding of the long-term outcomes and factors influencing continuity and changes in obsessive–compulsive symptoms over time is needed. The current study addressed three related aims. First, given the limited research into the stability of OCD symptoms in large community samples of young people, we aimed to investigate the extent to which OCB is stable across development. More specifically, we explored the persistence of OCB severity over a 12-year period from early childhood (age 4 years) to adolescence (age 16 years). Second, we tested the extent to which genetic and environmental factors determine the stability of OCB over time. Third, we investigated the extent to which genetic and environmental effects on OCD vary over time. We tested two possible models: that a single set of genetic/environmental risk factors make an impact on OCB across developmental stages; or that the influence of genetic/environmental factors on OCB varies over time through genetic innovation and attenuation.

## Method

### Participants

Participants were drawn from the Twins Early Development Study (TEDS), a large longitudinal study of twins born in England and Wales between 1994 and 1996 (Trouton *et al.* 2002). Details of recruitment and the sample are provided elsewhere (Haworth *et al.* 2013). TEDS was approved by the Institute of Psychiatry Ethics Committee and informed consent was obtained from participants. Zygosity of twins was determined using parental ratings of physical similarity (Price *et al.* 2000) and supplemented with DNA genotyping in a subsample for whom parent ratings were ambiguous. Participants were excluded if they did not provide consent, if they had severe medical disorders, experienced severe perinatal complications or if their zygosity was unknown (6.6% of the overall sample).

All procedures contributing to this work comply with the ethical standards of the relevant national and institutional committees on human experimentation and with the Helsinki Declaration of 1975, as revised in 2008.

The current study included data from four waves of TEDS: age 4, 7, 9 and 16 years (see Table 1 for mean ages). The sample size at each time point, separated by zygosity, is shown in Table 1. In total, 3224 individuals completed OCB items at all four time points. Individuals with data at all ages scored slightly lower on the OCB scale at age 4 years (mean = 2.48 *v.* 2.59). Although this difference was significant (*t* = 3.22, *p* < 0.001), the magnitude of the difference was small and not clinically meaningful. Drop-out rates were significantly higher for males than females (χ^2^ = 54.66, *p* < 0.001).

**Table 1.** Descriptive statistics and phenotypic correlations

	Age 4 years	Age 7 years	Age 9 years^a^	Age 16 years
Sample size, *n*				
MZ	5072	5202	2421	3497
DZ	9671	9216	4103	6150
Total	14 743	14 418	6524	9647
Descriptive statistics				
Mean age, years (s.d.)	4.04 (0.13)	7.07 (0.25)	9.02 (0.29)	16.32 (0.68)
Cronbach's *α* for OCB scale	0.59	0.59	0.62	0.61
Mean OCB score (s.d.)	2.56 (1.75)	1.89 (1.56)	0.94 (1.13)	0.82 (1.26)
Phenotypic correlations				
Age 4 years				
*r*	1			
*n*	14 743			
Age 7 years				
*r* (95% CI)	0.45 (0.43–0.46)	1		
*n*	11 276	14 418		
Age 9 years				
*r* (95% CI)	0.35 (0.32–0.37)	0.44 (0.42–0.46)	1	
*n*	5409	5771	6524	
Age 16 years				
*r* (95% CI)	0.23 (0.21–0.26)	0.32 (0.30–0.34)	0.37 (0.34–0.40)	1
*n*	7891	8053	4054	9647

MZ, Monozygotic; DZ, dizygotic; s.d., standard deviation; OCB, obsessive–compulsive behaviour; CI, confidence interval.

a At age 9 years, the study was restricted to just two out of three cohorts, resulting in a reduced sample size.

### Measures

The measure of OCB severity was extracted from the parent-report Anxiety-Related Behaviour Questionnaire (ARBQ) (Eley *et al.* 2003). The ARBQ includes items to assess anxiety symptoms across diagnostic categories, including OCD. The questionnaire is not a diagnostic instrument but shares similarities with other widely used screening measures of child anxiety disorders (e.g. the Spence Children's Anxiety Scale; Spence *et al.* 2003) and OCD (e.g. the Short OCD Screener; Uher *et al.* 2007). All ARBQ items are rated on a three-point scale (0 = never, 1 = sometimes, 2 = often).

Principal components analysis (PCA) was used to examine the factor structure of the ARBQ items at the ages of 7 and 16 years, in order to identify the items loading onto an OCB subscale. These analyses were not performed at the ages of 4 and 9 years given that the factor structure and OCB subscale had previously been established in the same sample (Eley *et al.* 2003; Hallett *et al.* 2009)†. In line with previous methods (Hallett *et al.* 2009), the PCA included an oblique rotation (Direct Oblimin command) and the number of factors was initially determined by using eigenvalues greater than 1. The PCA revealed seven underlying factors at age 7 years, and four underlying factors at age 16 years (see online Supplementary Tables S1 and S2). However, at age 7 years the sixth and seventh factor did not represent a meaningful construct and therefore the PCA was repeated with five fixed factors (this did not affect items loading on OCB). At both the ages of 7 and 16 years, the third factor that emerged was comparable with the OCB factor identified previously (Eley *et al.* 2003; Hallett *et al.* 2009). In summary, the OCB subscale included: four items at age 4 years (Eley *et al.* 2003); three items at age 7 years; two items at age 9 years (Hallett *et al.* 2009); and four items at age 16 years. The internal consistency and mean scores for the OCB subscale at each age are presented in Table 1.

### Analyses

#### Phenotypic analyses

Composite scores for the OCB subscale at each age were calculated. In a small proportion of cases (1.2% at age 4 years, 0.3% at age 7 years, 0.6% at age 9 years and 1.0% at age 16 years) there were missing OCB items. In these cases, the missing OCB item was calculated using the participant's mean OCB subscale score, with one missing item allowed per participant per time point. Within-subject phenotypic correlations over time were calculated to determine the stability of OCB. In order to obtain an initial gauge of heritability, cross-twin correlations were calculated at each age for monozygotic (MZ) and dizygotic (DZ) twins (e.g. twin 1 at age 4 years with twin 2 at age 4 years). Similarly, in order to obtain an initial gauge of genetic influences on OCB stability, cross-twin cross-age correlations were calculated (e.g. twin 1 at age 4 years with twin 2 at age 7 years).

#### Model fitting

The twin design compares the degree of phenotypic similarity between MZ twins, who share 100% of their genes, with DZ twins, who share 50% of their segregating genes on average. Within-pair correlations for MZ and DZ twins are compared in order to estimate the effects of: additive genetic factors (A); shared environment (C), which is defined as aspects of the environment that contribute to phenotypic similarity between siblings; and non-shared environment (E), which is defined as environmental factors that give rise to phenotypic differences between siblings. Greater within-pair correlations among MZ twins *versus* DZ twins indicate genetic influences on the phenotype of interest. Within-pair similarity that is not accounted for by genetic factors is attributed to shared environmental effects. Non-shared environmental effects are estimated from the within-pair differences between MZ twins; this also includes measurement error. Where correlations between DZ twins are less than half that of MZ twins, dominant genetic effects (D) are tested using an ADE model (see Plomin *et al.* 2013).

Twin analyses were conducted using OpenMx within R (Boker *et al.* 2011), a structural equation modelling package for the analysis of genetically informative data that controls for non-independence of family member data. Variables were regressed for age and sex, as is standard practice for quantitative genetic model fitting (McGue & Bouchard, 1984). Raw scores were normalized using Van der Warden transformations to correct for skew. Models were fitted using raw data maximum likelihood. The fit statistic provided by Mx for raw data modelling is minus twice the log likelihood (−2LL) of the observations. This is not an overall measure of fit but provides a relative measure of fit, because differences in −2LL between models are distributed as χ^2^. Therefore, the −2LL was compared with that of a saturated model in order to examine the overall fit of the genetic model. The fit of each sub-model was assessed by χ^2^ difference tests and the Akaike's information criterion (AIC = χ^2^ – 2 degrees of freedom), with lower χ^2^ values and more negative AIC values suggesting a better fit. When the difference between the AIC of two models was less than 10, the more parsimonious model was selected (Wagenmakers & Farrell, 2004).

#### Multivariate models

**Fig. 1.**
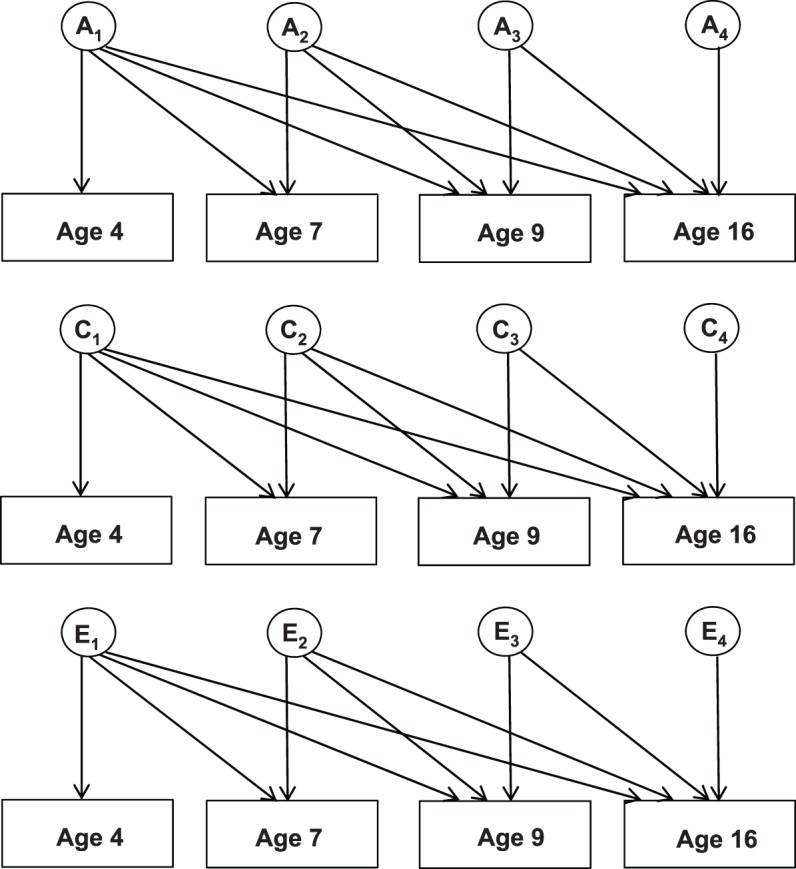
Cholesky decomposition ACE model. A, Additive genetic parameters; C, shared environment parameters; E, non-shared environment parameters.

We examined additive genetic (A), shared environment (C), dominant genetic (D) and non-shared environment (E) influences on OCB across development using a multivariate Cholesky decomposition. The Cholesky decomposition included the four OCB variables in temporal order (i.e. OCB at age 4, 7, 9 and 16 years). It assumes four distinct sets of genetic and environmental effects emerging at each time point (genetic and environmental innovation), which can exert influence on the variables at subsequent time points (see Fig. 1). A_1_, C_1_ and E_1_ are common factor influences on the first variable (OCB at age 4 years) that can also influence the remaining three variables (OCB at ages 7, 9 and 16 years). A_2_, C_2_ and E_2_ are new influences on the second variable and can also influence variables at the two later time points over and above the influences accounted for by A_1_, C_1_ and E_1_. Similarly, A_3_, C_3_ and E_3_ influence the third variable and can also influence the fourth variable over and above the influences accounted for by A_1_, C_1_ and E_1_, and A_2_, C_2_ and E_2_. Finally, A_4_, C_4_ and E_4_ are unique influences emerging at the latest time point to influence the fourth variable only. Total A, C and E effects on OCB at each age can be obtained by summing all paths to that measure.

The Cholesky decomposition can be represented as a multivariate correlated factors solution. It assumes that each variable has unique A, C and E influences, and that these trait-specific influences are correlated with the A, C and E influences on other traits. The proportions of the phenotypic correlations accounted for by A, C and E influences were also calculated.

The relative fit of ACE and ADE models, with and without sex differences, was compared, and all models were compared with the saturated model. Quantitative and qualitative sex differences were tested to see whether males and females differ in magnitude or nature of genetic and environmental influences, respectively. Scalar sex differences were tested which assessed whether males and females showed differences in variance.

## Results

### Phenotypic analyses

The first aim was to establish the stability of OCD over time. Within-person phenotypic correlations over time were calculated. As shown in Table 1, overall the correlations indicated a moderate degree of stability in the expression of OCB. The strength of correlation reduced with increasing time intervals; the correlation was highest between the ages of 7 and 9 years (*r* = 0.45) and weakest between the ages of 4 and 16 years (*r* = 0.23).

### Multivariate analysis

Multivariate analyses were conducted in order to address the second and third aims of the study, which were to establish the genetic and environmental influences on OCB stability and to test the extent to which these influences remained stable or changed over time. At each age, the within-age and across-ages twin correlations were higher among MZ than DZ pairs, indicating genetic influences on the OCB phenotype within time and longitudinally (see online Supplementary Table S3). In a number of instances, the MZ correlation was more than twice that of the DZ correlation, indicating possible dominant genetic effects. We therefore compared the relative fit of the ACE and ADE models; both models were tested with and without sex differences. Although the models including quantitative sex differences provided a better fit, the pattern of results was the same for both sexes and therefore a scalar model was selected. The scalar variable was included at all ages to account for different variances in OCB scores between males and females. The overall best-fitting model, according to the lowest AIC value, was the ACE model. However, because some of the DZ correlations were less than half of the MZ correlations, A should be interpreted more broadly as both additive and dominant genetic effects. No parameters could be dropped from the model without deteriorating the fit. Multivariate model comparisons are presented in the online Supplementary Material (Table S4), along with the results of the ACE multivariate analyses with quantitative sex differences (online Supplementary Tables S5 and S6).

Parameter estimates for the proportion of variance and covariance in OCB accounted for by A, C and E within and across ages are presented in Table 2. As shown along the diagonal, moderate genetic influences on OCB were found at each age, ranging from 0.33 (at age 16 years) to 0.62 (at age 7 years). There was also a substantial influence of non-shared environment, ranging from 0.32 (at age 9 years) to 0.42 (at age 16 years). The effect of shared environment was not significant at age 4 years, and small at ages 4, 9 and 16 years. The off-diagonal shows the effects of A, C and E on the covariance in OCB over time (i.e. OCB stability). Large genetic influences on OCB stability were found, ranging from 0.59 (age 7 to 16 years) to 0.80 (age 4 to 9 years). The effect of shared environment on OCB stability was generally not significant. However, significant small non-shared environmental influences on stability were found at all ages, ranging from 0.12 (age 4 to 16 years) to 0.30 (age 7 to 9 years).

**Table 2.** Proportion of phenotypic variance and covariance accounted for by A, C and E^a^

	Age 4 years	Age 7 years	Age 9 years	Age 16 years
Proportion of phenotypic variance and covariance accounted for by A				
Age 4 years	0.59 (0.57–0.61)			
Age 7 years	0.75 (0.70–0.79)	0.61 (0.57–0.63)		
Age 9 years	0.80 (0.70–0.91)	0.70 (0.66–0.86)	0.54 (0.46–0.61)	
Age 16 years	0.78 (0.68–0.97)	0.59 (0.43–0.75)	0.72 (0.57–1.00)	0.33 (0.26–0.41)
Proportion of phenotypic variance and covariance accounted for by C				
Age 4 years	0.00 (0.00–0.01)			
Age 7 years	0.01 (0.00–0.04)	0.02 (0.01–0.04)		
Age 9 years	0.00 (0.00–0.09)	0.00 (0.00–0.02)	0.14 (0.09–0.21)	
Age 16 years	0.10 (0.00–0.25)	0.17 (0.04–0.30)	0.00 (0.00–0.12)	0.25 (0.19–0.31)
Proportion of phenotypic variance and covariance accounted for by E				
Age 4 years	0.41 (0.38–0.43)			
Age 7 years	0.24 (0.20–0.28)	0.38 (0.36–0.40)		
Age 9 years	0.21 (0.15–0.27)	0.30 (0.26–0.35)	0.32 (0.29–0.35)	
Age 16 years	0.12 (.03–0.20)	0.24 (0.18–0.30)	0.28 (0.21–0.36)	0.42 (0.39–0.45)

Data are given as proportion (95% confidence interval).

A, Additive genetic parameters; C, shared environment parameters; E, non-shared environment parameters.

a The same pattern of results was found when we replicated the analyses using a stable index of obsessive–compulsive behaviour (i.e. the same three items at each age).

**Fig. 2.**
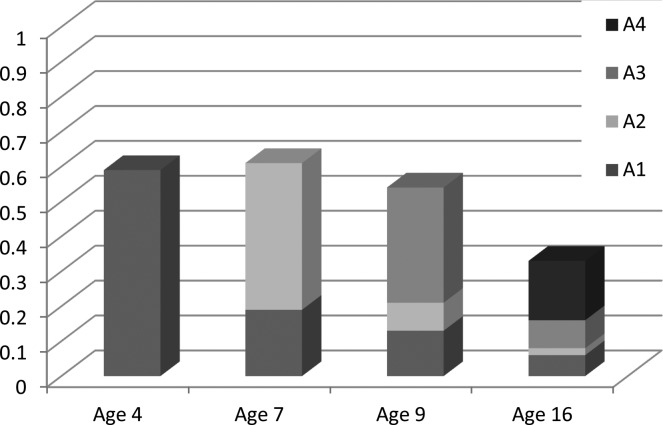
The proportion of total variance in obsessive–compulsive behaviour (OCB) symptoms accounted for by stable and new genetic factors across development. The *y*-axis represents the total phenotypic variance. A1 represents the first genetic factor that is evident at age 4 years. A2 represents the second genetic factor that emerges at age 7 years. A3 is the third genetic factor that arises at age 9 years, and A4 is the fourth genetic factor that emerges at age 16 years. The total genetic influence on OCB at each age is the sum of all factors (A1, A2, A3 and A4) at that time point.

The Cholesky decomposition informs us about the effect of stable and new genetic and environmental factors across the four ages. Parameter estimates are presented in Table 3. Results indicate genetic continuity, whereby genetic factors influencing OCB at any one age continue to affect OCB at subsequent ages. However, their influence declines over time, indicating genetic attenuation. For example, the first set of genetic factors (A_1_) accounted for 59% of the variance in OCB at age 4 years, but reduced to 19% by age 7 years, 13% by age 9 years and 6% by age 16 years. Furthermore, substantial new genetic factors emerged at each age, indicating genetic innovation. The new genetic effects that emerged at each age accounted for more than 50% of the total genetic influence on OCB at that time. The effect of non-shared environment was largely age specific. For example, the non-shared environmental factors influencing OCB at age 4 years (E_1_) had a negligible effect on OCB at age 7 years, and no significant effect at the ages of 9 and 16 years. Fig. 2 illustrates the genetic attenuation and innovation effects. Of note, the same pattern of results was found when we replicated the analyses using a stable index of OCB (i.e. the same three items at each age), demonstrating that the attenuation and innovation effects were not due to variation in the item content of the OCB measure at each age (see online Supplementary Table S7).

**Table 3.** Cholesky decomposition results^a^

	A1	A2	A3	A4
Age 4 years	0.59 (0.57–0.62)			
Age 7 years	0.19 (0.16–0.22)	0.42 (0.38–0.45)		
Age 9 years	0.13 (0.09–0.17)	0.08 (0.04–0.13)	0.33 (0.26–0.40)	
Age 16 years	0.06 (0.03–0.09)	0.02 (0.00–0.05)	0.08 (0.02–0.17)	0.17 (0.08–0.26)
	C1	C2	C3	C4
Age 4 years	0.00 (0.00–0.01)			
Age 7 years	0.01 (0.00–0.04)	0.01 (0.00–0.04)		
Age 9 years	0.00 (0.00–0.19)	0.14 (0.00–0.20)	0.00 (0.00–0.20)	
Age 16 years	0.25 (0.00–0.31)	0.00 (0.00–0.28)	0.00 (0.00–0.21)	0.00 (0.00–0.20)
	E1	E2	E3	E4
Age 4 years	0.41 (0.38–0.43)			
Age 7 years	0.03 (0.02–0.04)	0.35 (0.33–0.37)		
Age 9 years	0.01 (0.00–0.02)	0.04 (0.03–0.05)	0.27 (0.25–0.29)	
Age 16 years	0.00 (0.00–0.01)	0.01 (0.01–0.02)	0.02 (0.01–0.04)	0.38 (0.35–0.41)

Data are given as parameter estimate (95% confidence interval).

A, Additive genetic parameters; C, shared environment parameters; E, non-shared environment parameters.

a Note that some of the zeros in the tables are due to rounding to two decimal places. The same pattern of results was found when we replicated the analyses using a stable index of obsessive–compulsive behaviour (i.e. the same three items at each age).

## Discussion

This is the first study to examine the stability of severity of OCB from early childhood to late adolescence in a large community sample. In relation to our first aim, we found that OCB severity was moderately stable across development. OCB was more likely to persist over shorter time intervals and relatively less stable over greater periods of time. These findings are in line with the results of the only previous study that has examined OCB longitudinally in an unselected community sample of children (van Grootheest *et al.* 2007). Our findings are also broadly consistent with outcomes found in adult community samples (van Grootheest *et al.* 2009).

The second aim was to examine the influences of genetic and environmental factors on the stability of childhood OCB. We found that persistence of OCB is largely driven by genetic factors, with up to 80% of stability being accounted for by genetic influences. Non-shared environmental factors also contributed to the stability of OCB over time but the effect was relatively small. These findings are broadly in keeping with previous research showing that genetic factors play a larger role in determining the stability of obsessive–compulsive symptoms over time than environment (van Grootheest *et al.* 2007, 2009). Genetic influences on OCB persistence have been estimated to be approximately 40% in child samples (van Grootheest *et al.* 2007) and 75% in adult samples (van Grootheest *et al.* 2009). Our findings are consistent with the results of the adult study and suggest that the extent to which genes influence the continuity of OCB in childhood may be greater than previously thought.

The final aim was to explore the pattern of genetic and environmental influences on the severity of OCB across development. We found that the genetic factors influencing OCB at any one age continued to exert an effect on the expression of OCB at subsequent ages, suggesting that there is some continuity in the genes underlying OCB at different ages, which is in keeping with a number of previous studies in anxiety and depression (O'Connor *et al.* 1998; Trzaskowski *et al.* 2012; Zavos *et al.* 2012). However, we also found evidence for significant genetic attenuation, whereby the effect of a specific set of genes on OCB diminished over time, and genetic innovation, in which new genes appeared to ‘come on line’ at different ages. In other words, our findings suggest that the genetic architecture of OCB may vary across development. These findings are in line with previous research showing genetic attenuation and innovation in relation to internalizing disorders (Scourfield *et al.* 2003; Lau & Eley, 2006; Kendler *et al.* 2008*a*, *b*). These data are also consistent with observations that OCD is a dynamic phenotype, and typically follows a waxing and waning course, with symptoms often changing over time (Rettew *et al.* 1992; Mataix-Cols *et al.* 2002). Interestingly, previous studies in OCB have found smaller genetic innovation than were obtained in the current study (van Grootheest *et al.* 2007, 2009). One possible explanation for this apparent discrepancy is that genetic innovation may be more evident at certain stages of development. For example, genetic influences on depression may be more stable in adulthood than adolescence (Kendler *et al.* 1993; Lau & Eley, 2006). In the current study we investigated OCB over a particularly broad period of development and one that is associated with significant biological changes.

With respect to non-shared environment, we found that effects were largely age-specific, with little continuity of effects across development. An inherent limitation of twin modelling is that estimates of the non-shared environment are conflated by measurement error. It is possible that measurement error varied at each age due to the changing composition of the OCB measures, and it could be argued that this gave rise to the apparent attenuation and innovation effects. However, this is unlikely given that the same pattern of results was found when the analyses were repeated using a stable index of OCB (see online Supplementary Table S7). The finding that non-shared environmental influences are age-specific may reflect the fact that many unique environmental experiences are often time specific (e.g. bullying). While they may contribute to OCB in the short term, our findings suggest that they do not have a lasting effect on OCB. Similarly, previous studies have found non-shared environmental influences on anxiety to be time specific (Kendler *et al.* 2008*b*; Trzaskowski *et al.* 2012; Zavos *et al.* 2012).

The current study has a number of important clinical and theoretical implications. First, our findings demonstrate that severity of OCB is moderately stable across childhood. On the one hand, this can be viewed as providing a relatively optimistic message, in that OCB is not necessarily chronic. On the other hand, our findings show that compulsive behaviours emerging as early as 4 years of age can persist up to late adolescence. If these findings extend to clinically significant obsessive–compulsive symptoms, it would highlight the importance of early detection and intervention. Few studies have evaluated the treatment of OCD in young children (Comer *et al.* 2014; Freeman *et al.* 2014; Lewin *et al.* 2014), and further research is needed in this field. Second, our findings suggest that unique environmental experiences do not generally have an enduring effect on OCB traits, but rather their effects tend to be age specific. This finding supports the use of interventions that focus on ‘the here and now’ rather than past experiences, such as cognitive behaviour therapy, which is recommended as a first-line treatment for paediatric OCD (National Institute for Health and Clinical Excellence, 2005; Geller & March, 2012). Third, our novel finding that genetic effects on the severity of OCB are developmentally dynamic has crucial implications for molecular genetic research. Our findings suggest that molecular genetic studies of OCD should be age sensitive, rather than assuming that the same genes influence OCD at different ages and pooling data from participants across a wide range of ages. In this vein, gene–age interactions have been found in the field of physical health, including in relation to blood pressure regulation (Simino *et al.* 2014), lipid levels (Dumitrescu *et al.* 2011) and obesity (Lasky-Su *et al.* 2008).

The current study has a number of strengths, including the large sample size and the long follow-up period. However, the results must also be considered in the context of a number of limitations. First, the measure of OCB used only included two to four items, and is therefore likely to have captured a narrow range of the OCD phenotype. Of particular note, the items included focused solely on compulsive behaviours and did not encompass obsessional thoughts. Furthermore, the OCB subscales had relatively low levels of internal consistency, indicating considerable heterogeneity between items. This may reflect the small number of items included, and future studies should seek to replicate the current findings using a more robust measure of OCB. Second, the OCB measure used is not a diagnostic instrument and it cannot be assumed that the current findings would necessarily apply to diagnosable OCD. However, previous research supports the notion that OCD is a dimensional, not categorical, construct thereby validating research in analogue samples (Abramowitz *et al.* 2014). Third, the current study only included parent-report OCB. It will be important for future research to include child-report measures, particularly given that the agreement between child- and parent-report of OCD symptoms has been shown to be relatively poor (Canavera *et al.* 2009). On the other hand, child-report scales of psychopathology are typically only validated for use from 7 years onwards (e.g. Saylor *et al.* 1984). Children are unlikely to be able to complete self-report scales before this age, which would have precluded the possibility of having a child-report measure at every time point in the current study. Lastly, the current study has a number of limitations that are inherent to twin design studies as previously described (Plomin *et al.* 2013).

Notwithstanding these limitations, this is the first study to examine the genetic and environmental influences on OCB severity over a 12-year period from childhood to adolescence. Our results suggest that the persistence of OCB over time is largely driven by genetic factors. However, genetic influences on OCB are not stable but rather the genetic architecture of OCB appears to change across development. This could potentially explain temporal fluctuations in symptoms that are common to the disorder.

## Footnotes

† The notes appear after the main text.

## Supplementary material

For supplementary material accompanying this paper visit http://dx.doi.org/10.1017/S0033291714002761
